# Once-weekly Somapacitan is Effective and Well Tolerated in Adults with GH Deficiency: A Randomized Phase 3 Trial

**DOI:** 10.1210/clinem/dgaa049

**Published:** 2020-02-05

**Authors:** Gudmundur Johannsson, Murray B Gordon, Michael Højby Rasmussen, Ida Holme Håkonsson, Wolfram Karges, Claus Sværke, Shigeyuki Tahara, Koji Takano, Beverly M K Biller

**Affiliations:** 1 Sahlgrenska University Hospital, Göteborg, Sweden; 2 Allegheny General Hospital, Pittsburgh, PA; 3 Novo Nordisk A/S, Søborg, Denmark; 4 Division of Endocrinology and Diabetes, RWTH Aachen University, Aachen, Germany; 5 Nippon Medical School, Tokyo, Japan; 6 Department of Endocrinology, Diabetes and Metabolism, Kitasato University, Tokyo, Japan; 7 Neuroendocrine Unit, Massachusetts General Hospital, Boston, MA

**Keywords:** somapacitan, long-acting growth hormone, adult growth hormone deficiency, body composition, hypopituitarism, REAL 1

## Abstract

**Context:**

Growth hormone (GH) replacement requires daily GH injections, which is burdensome for some adult patients with GH deficiency (AGHD).

**Objective:**

To demonstrate efficacy and safety of somapacitan, a once-weekly reversible albumin-binding GH derivative, versus placebo in AGHD.

**Design:**

Randomized, parallel-group, placebo-controlled (double-blind) and active-controlled (open-label) phase 3 trial, REAL 1 (NCT02229851).

**Setting:**

Clinics in 17 countries.

**Patients:**

Treatment-naïve patients with AGHD (n = 301 main study period, 272 extension period); 257 patients completed the trial.

**Interventions:**

Patients were randomized 2:2:1 to once-weekly somapacitan, daily GH, or once-weekly placebo for 34 weeks (main period). During the 52-week extension period, patients continued treatment with somapacitan or daily GH.

**Main outcome measures:**

Body composition measured using dual-energy x-ray absorptiometry (DXA). The primary endpoint was change in truncal fat percentage to week 34. Insulin-like growth factor 1 (IGF-I) standard deviation score (SDS) values were used to dose titrate.

**Results:**

At 34 weeks, somapacitan significantly reduced truncal fat percentage (estimated difference: −1.53% [−2.68; −0.38]; *P* = 0.0090), demonstrating superiority compared with placebo, and it improved other body composition parameters (including visceral fat and lean body mass) and IGF-I SDS. At 86 weeks, improvements were maintained with both somapacitan and daily GH. Somapacitan was well tolerated, with similar adverse events (including injection-site reactions) compared with daily GH.

**Conclusions:**

In AGHD patients, somapacitan administered once weekly demonstrated superiority over placebo, and the overall treatment effects and safety of somapacitan were in accordance with known effects and safety of GH replacement for up to 86 weeks of treatment. Somapacitan may provide an effective alternative to daily GH in AGHD. A short visual summary of our work is available (1).

Growth hormone (GH) deficiency in adults (AGHD) is characterized by abdominal obesity, reduced lean body mass, fatigue, impaired psychosocial function, reduced aerobic exercise capacity, osteopenia, and elevated levels of circulating cardiovascular risk biomarkers (2, 3). Untreated AGHD has been associated with premature cardiovascular morbidity and mortality (2, 4). These features may be reversed or improved with the use of ongoing GH replacement (3), and meta-analyses have confirmed the beneficial effects of GH replacement on body composition, cardiac function, and bone mineral density (2), although, to date, GH replacement has not been definitively shown to reduce mortality. Clinical guidelines recommend GH replacement as a component of the care of hypopituitary patients (5–7).

GH replacement currently requires daily subcutaneous injections. A long-term or lifelong daily injection regimen can be burdensome for patients with AGHD, even though GH is administered with fine needles to minimize injection pain; thus treatment fatigue is observed and, consequently, compliance and adherence may be reduced (8). Furthermore, in clinical practice, patients with AGHD may receive no or inadequate GH replacement due to a number of factors, including the need for other concomitant treatments and an unwillingness to add to the patient’s treatment burden. The use of long-acting GH formulations with decreased injection frequency could potentially reduce the burden of treatment and treatment fatigue in hypopituitary patients and may thus lower the barrier to initiating replacement therapy.

Safety and tolerability are particularly important in view of the potentially lifelong duration of GH replacement therapy in AGHD. Previous studies in AGHD have reported similar safety for long-acting or sustained-release GH preparations compared with daily administration in studies lasting 32 weeks (9) and 26 weeks (10). Neither compound is commercially available in the US or Europe.

Somapacitan (Novo Nordisk A/S, Denmark) is a novel once-weekly reversible albumin-binding GH derivative, with a small albumin-binding moiety (1.2 kDa) attached to the GH molecule. This facilitates reversible binding to circulating endogenous albumin, thereby reducing the clearance and extending the half-life of somapacitan, allowing once-weekly administration. This is a well-established technique that has been used successfully to extend the half-lives of insulin detemir (11) and the glucagon-like peptide-1 (GLP-1) agonists liraglutide (12) and once-weekly semaglutide (13).

Once-weekly somapacitan has been shown in previous trials to be well tolerated in healthy adults (14), and in adults and children with growth hormone deficiency (GHD) (15, 16), and demonstrated similar efficacy to daily GH in children with GHD (17). No safety or tolerability issues with once-weekly somapacitan were identified in a 26-week trial in patients with AGHD previously treated with daily GH (REAL 2); furthermore, somapacitan was reported to be more convenient than once-daily GH in these patients (18). However, to date, no trials of the efficacy of somapacitan in AGHD (other than treatment satisfaction) have been reported.

The present report describes a large multinational, randomized, phase 3 trial (REAL 1) that examined the efficacy and safety of somapacitan administered once-weekly in patients with AGHD, naïve to GH replacement therapy or off GH therapy for at least 6 months (NCT02229851). The primary objective was to show superiority of somapacitan versus placebo. A secondary comparison of somapacitan with once-daily GH was also made, to compare efficacy and safety.

## Materials and Methods

### Study design

**Figure 1.**
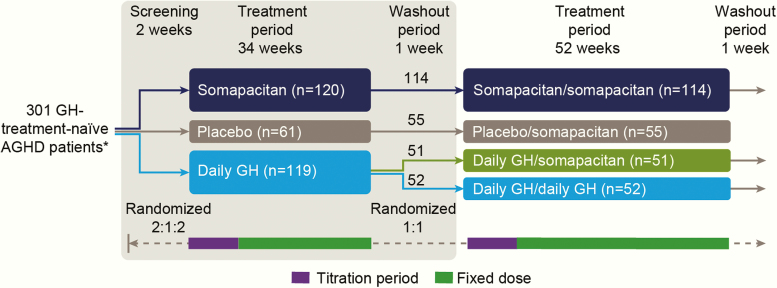
Trial design. *Numbers in the treatment boxes show patients exposed to treatment. One patient in the somapacitan group did not receive any treatment and was not included in any analyses. The grey box indicates the main period of the trial. Purple bars indicate titration periods and green bars, fixed-dose treatment periods. Time axis is not to scale. Abbreviations: AGHD, adult growth hormone deficiency

REAL 1 was a multicenter, multinational, randomized, parallel-group, placebo- and active-controlled phase 3 trial to compare the efficacy and safety of once-weekly somapacitan with once-weekly placebo for up to 34 weeks of treatment (main period) in AGHD patients. An open-label daily GH (somatropin; Norditropin FlexPro; Novo Nordisk A/S, Denmark) arm was also included for comparison. The main period of the trial was followed by a 52-week extension period (Fig. 1), in order to evaluate the efficacy and safety of somapacitan for up to 86 weeks of treatment.

The protocol was approved by local and national ethics committees, as appropriate, and conducted in accordance with the International Conference on Harmonisation guidelines for Good Clinical Practice (19) and the Declaration of Helsinki (20). Written informed consent was obtained from all patients prior to inclusion.

The main trial period consisted of 8 weeks of dose titration followed by 26 weeks of fixed-dose treatment, followed by a 1-week washout. Patients were randomized 2:1:2 to receive once-weekly somapacitan, once-weekly placebo, or daily GH (Fig. 1). The main period was double-blind with respect to once-weekly somapacitan and placebo, but open-label with respect to daily GH, and the investigators and trial sites remained blinded throughout the trial. The extension period was open-label. Randomization was stratified according to region (Japan/all other countries), sex, and diabetic status, and was performed via a trial-specific, web-based interactive voice/web response system.

Patients completing the main period were eligible to be enrolled into a 52-week extension. After a 1-week washout at the end of the 34 weeks, patients receiving somapacitan continued treatment (somapacitan/somapacitan group), those receiving placebo were switched to somapacitan (placebo/somapacitan group), and those receiving daily GH were randomized 1:1 to continue with daily GH (daily GH/daily GH group) or receive once-weekly somapacitan (daily GH/somapacitan group) (Fig. 1).

### Patients

The trial was conducted at 92 sites from October 2014 to May 2017 (main period) and to May 2018 (extension period) in the following 17 countries: Australia, Germany, India, Israel, Japan, Latvia, Lithuania, Malaysia, Poland, Romania, Russian Federation, South Africa, Sweden, Turkey, Ukraine, the United Kingdom, and the United States.

Inclusion criteria were as follows: patients aged 23 to 79 years, with a diagnosis of adult- or childhood-onset AGHD confirmed as follows: peak GH response < 3 ng/mL in the insulin tolerance test (ITT) or glucagon test, or peak GH response < cutoff values based on body mass index in the GH-releasing hormone (GHRH) + arginine test, or 3 or more pituitary hormone deficiencies and insulin-like growth factor 1 (IGF-I) standard deviation scores (SDS) < −2.0. For Japan only, patients with a history of childhood-onset GHD needed to meet at least 2 of the following criteria (1 criterion if they had organic disease with multiple pituitary hormone deficiency [MPHD]): peak GH response ≤ 1.8 ng/mL in the ITT, or ≤ 1.8 ng/mL in the glucagon test, or ≤ 9 ng/mL in the GH-releasing peptide-2 test. Patients with adult-onset AGHD had to satisfy at least 2 of the above criteria for isolated GHD or at least 1 criterion if they had organic disease with MPHD.

Further criteria were: naïve to GH treatment or no exposure to GH therapy/GH secretagogues (except if used to diagnose AGHD) for ≥ 180 days prior to randomization; IGF-I SDS at screening < −0.5 relative to the mean of the age- and sex-specific normal ranges used by the central laboratory; any replacement therapies for other hormone deficiencies adequate and stable for ≥ 90 days prior to randomization; male patients with adequate endogenous or replaced testosterone level and all patients with serum levels of free T4 within normal limits according to the central laboratory measurements; adequate adrenal function, confirmed with an ACTH stimulation test or ITT within 90 days prior to randomization, or adequate and stable cortisol replacement therapy (as judged by the investigator) for ≥ 90 days prior to randomization.

Patients with diabetes mellitus had to meet the following additional criteria: diabetes mellitus diagnosed clinically ≥ 6 months prior to screening; on stable oral antidiabetic treatment, unchanged for ≥ 90 days prior to screening; no history of use of injectable anti-diabetic agents; HbA_1c_ < 7.0% at screening according to the central laboratory; no diabetes-related comorbidities (as judged by the investigator) at screening; and no evidence of proliferative retinopathy or severe nonproliferative diabetic retinopathy on fundus photography performed ≤ 90 days prior to randomization.

Key exclusion criteria are shown in Table 1.

**Table 1.** Exclusion Criteria.

• Known or suspected hypersensitivity to trial product(s) or related products
• For female patients, pregnancy, breast-feeding, or the potential to become pregnant (specific criteria varied in some countries). Males of reproductive age were excluded if they or their partner(s) were not using adequate contraceptive methods
• Active malignant disease or history of malignancy, with some exceptions
• History of pituitary adenomas or other benign intracranial tumors, unless the tumor had been surgically removed >12 months before randomization or the patients had stable and clinically non-functioning adenomas for ≥3 years, with the most recent scan within 9 months prior to randomization
• Clinically significant renal or hepatic disease
• A history of positive tests for hepatitis B and/or C or for human immunodeficiency virus antibodies
• Acute severe illness associated with weight loss <180 days prior to randomization
• Active Cushing’s syndrome <24 months prior to randomization
• Heart insufficiency (NYHA class >2)
• A history of acromegaly
• Use of systemic corticosteroids other than in replacement doses within 90 days before randomization
• Mental incapacity/language barriers which precluded adequate participation

### Study drug administration and dose selection

Trial products were administrated by subcutaneous injections with equivalent pen devices based on the FlexPro platform. A NovoFine 32G Tip needle (in Japan, PenNeedle 32G) was used for all products. Patients were trained to inject themselves with trial drug by site staff and instructed to alternate between the abdomen and the thighs. Patients receiving somapacitan or placebo injected themselves once weekly in the morning. Patients receiving daily GH injected themselves daily in the evening, as is standard treatment practice, except during observed trial drug administration (where injections were done in the morning, at least 12 hours after injection the evening before). Initial doses of somapacitan prior to individual titration, presented as mg/week (mg/day), were set based on doses previously investigated in a multiple dose pharmacokinetic/pharmacodynamic trial in patients with AGHD (15). Starting doses were as follows: patients aged 23 to 60 years, 1.5 (0.214); patients aged > 60 years: 1.0 (0.143); and female patients on oral estrogen irrespective of age: 2.0 (0.286). Corresponding starting doses for daily GH were 0.2, 0.1, and 0.3 mg/day. Minimum and maximum doses were set to 0.1 mg and 8 mg weekly for somapacitan, and to 0.05 mg and 1.1 mg daily (1.0 mg in Japan) for daily GH.

**Figure 2.**
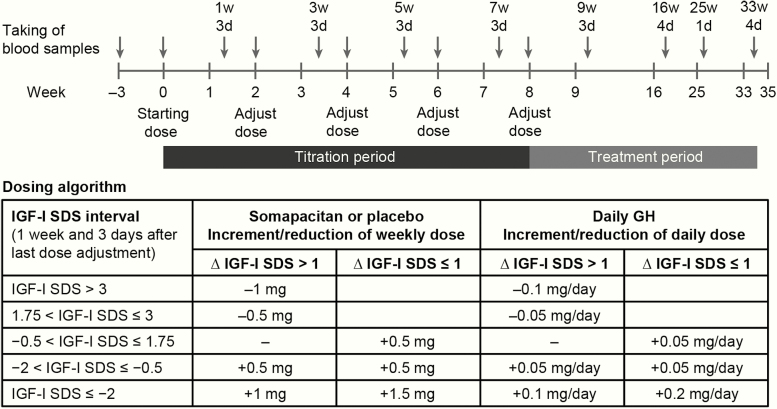
Blood sampling for IGF-I in the main study period and dose titration of somapacitan, placebo, and daily GH. Arrows indicate timing of blood sampling, which was performed before administration of drug (weeks 0, 2, 4, 6, 8) or between planned doses (weeks 1, 3, 5, 7, 9, 16, 25, and 33). For the extension period, titration followed a similar pattern, with week 35 corresponding to week 0. Blood sampling also followed a similar pattern up to 53w4d (corresponding to 16w4d) and continued at 64w1d, 75w4d, and 86w4d. Time axis is not to scale. Abbreviations: d, days; GH, growth hormone; IGF-I, insulin-like growth factor-I; SDS, standard deviation scores; w, weeks.

Doses were titrated according to an algorithm in order to achieve a steady state IGF-I SDS target of −0.5 to +1.75 (Fig. 2). The titration period allowed for 4 opportunities to adjust the dose, and at least 3 dose adjustment evaluations were required. Blood samples were taken 1 week and 3 days after the previous dose adjustment visit and dose adjustments were performed 4 days later (Fig. 2). After week 8, individual dose levels were fixed, but could be reduced at the investigator’s discretion for safety concerns. To avoid unblinding, dummy titration of placebo patients mirrored the titration of somapacitan patients.

### Efficacy assessments

The primary efficacy endpoint was change from baseline to week 34 in truncal fat percentage (defined as 100 times truncal fat mass divided by the sum of truncal fat mass and truncal lean body mass). Secondary endpoints included the change from baseline to week 86 in truncal fat percentage and changes from baseline to weeks 34 and 86 in dual-energy x-ray absorptiometry (DXA)-derived body composition measures (listed below) and in IGF-I SDS and insulin-like growth factor binding protein-3 (IGFBP-3) SDS. Changes in body weight, waist circumference, lipid profile, high-sensitivity C-reactive protein (hsCRP), and interleukin-6 were also assessed.

The following parameters were measured by DXA: truncal fat mass, total fat mass, lean body mass, truncal lean body mass, and appendicular skeletal muscle mass, all in grams. Android fat mass and gynoid fat mass in grams, and visceral fat in cm^2^, were measured if the scanner permitted. Percentage change from baseline in visceral fat was reported as a *post hoc* defined endpoint. Total body bone mineral content (BMC) and bone mineral density (BMD) were assessed at baseline and week 86 using DXA.

Three DXA scans were performed: at screening and at the end of each period. The imaging was performed in a standardized manner following a written guideline at all sites. Scans were read by staff at the central imaging laboratory who were blinded to treatment group. A cross calibration using a phantom was performed at least once at each site prior to the database lock of the main period.

Analyses of serum IGF-I and IGFBP-3 were performed by the central laboratory using commercially available assay kits (Immuno Diagnostic Systems immunoassay [ISYS assay]). IGF-I SDS and IGFBP-3 were calculated using the reference data published by Bidlingmaier and Friedrich, respectively (21, 22). Analyses for hematology, glucose metabolism, and biochemistry including lipids and hsCRP and were performed by standard procedures at the central laboratory.

### Safety assessments

Safety was assessed by the incidence of adverse events (AEs), which were summarized by treatment, Medical Dictionary for Regulatory Activities (MedDRA) system organ class, and MedDRA preferred term. Assessment of antibodies against somapacitan (somapacitan and placebo groups) or GH (daily GH group) was performed by the study sponsor using a validated anti-somapacitan or anti-human GH antibody-binding assay.

### Statistical analysis

The primary objective was to show superiority of somapacitan versus placebo on the primary endpoint, in other words, to confirm the hypothesis of a treatment difference on truncal fat percentage. Superiority of somapacitan over placebo was considered confirmed if the upper boundary of the two-sided 95% CI of the estimated treatment difference (ETD) (somapacitan—placebo) was below 0.

A secondary comparison of the primary endpoint, comparing somapacitan with daily GH, was used to assist in judging the clinical relevance of the ETD between somapacitan and placebo. As no difference was expected between somapacitan and daily GH, this was not designed as a confirmatory test and no hierarchical test strategy was constructed; therefore, no *P* value was calculated.

An analysis of covariance model (ANCOVA) was used to compare the changes from baseline to week 34 for DXA-derived measures (including the primary endpoint), waist circumference, and log-transformed lipid profile data. These analyses were conducted using a multiple imputation technique to deal with missing data, where the trajectory after a withdrawn participant’s last observation was imputed based on data from the placebo arm (on the assumption that withdrawn patients would be switched to no treatment after withdrawal). As only one confirmatory test was defined for the trial, no adjustment for multiplicity was needed; all other hypotheses were nonconfirmatory and/or supportive secondary endpoints and did not affect the significance level used for the confirmatory test; thus, *P* values were reported.

All other supportive secondary efficacy endpoints were analyzed with the use of a mixed model for repeated measurements (MMRM). From the statistical model (ANCOVA or MMRM), the ETDs at week 34 between somapacitan and placebo and between somapacitan and daily GH were estimated (termed *adjusted values*), and the corresponding 95% CIs and *P* values were calculated for each secondary endpoint. For these analyses, GHD onset type, sex, region, diabetes mellitus status, and interaction between sex, region, and diabetes mellitus were included as factors and baseline values as covariates in the model. Changes from baseline to end of week 86 in the above variables were analyzed using descriptive statistics and supplemented with an exploratory analysis based on MMRM analysis models similar to those used in the main trial period, in which the somapacitan/somapacitan and daily GH/daily GH arms were compared. Supportive secondary safety endpoints were analyzed using descriptive statistics.

Under the assumption of a true mean difference of 2.5% between somapacitan and placebo, a standard deviation of 4.5% for the primary endpoint and a dropout rate of 7%, with 50% of patients not completing the main trial contributing with postrandomization DXA data, it was estimated that including 112 patients in the somapacitan group and 56 patients in the placebo group would provide 89% power for detecting a difference in the primary endpoint between the 2 groups with a two-sided, 5% significance level. Adding the daily GH arm, a total of 280 patients needed to be included in the trial.

The full analysis set (FAS), used to evaluate efficacy endpoints, included all randomized subjects who received at least one dose of randomized treatment. In this study, the FAS and the safety analysis set (SAS), used to evaluate safety endpoints, were identical.

Treatment compliance was assessed using the recorded treatment doses in the patient’s diary. Adherence was calculated as the number of doses that patients reported taking multiplied by 100 and divided by the number of prescribed doses.

## Results

### Study population

**Figure 3.**
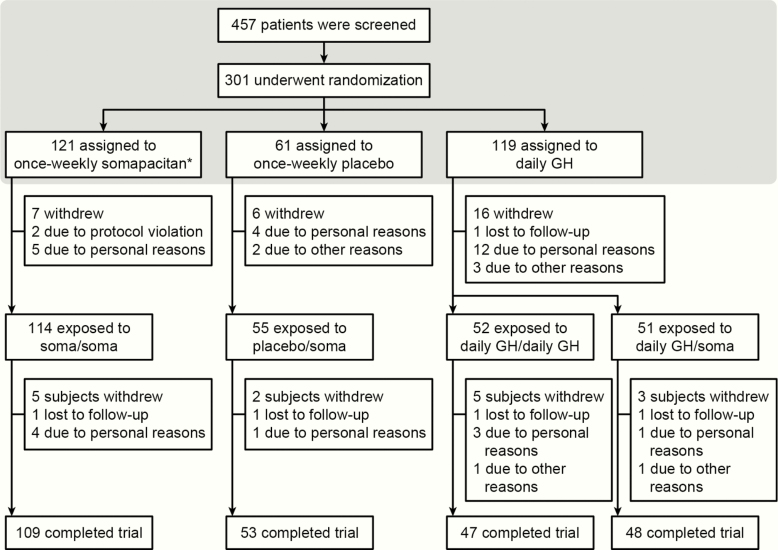
Patient disposition. *One patient in the somapacitan group was randomized but did not receive any trial drug (no reason was provided) and was therefore not included in any analyses. The shaded box represents the main period of the trial. Abbreviations: GH, growth hormone; soma, somapacitan

Of the 457 patients initially screened, 301 were randomized to receive once-weekly somapacitan (n = 121), once-weekly placebo (n = 61), or daily GH (n = 119) (Fig. 3). One patient in the somapacitan group did not receive any treatment. During the extension period, patients received treatment as follows: somapacitan/somapacitan (n = 114), daily GH/daily GH (n = 52), placebo/somapacitan (n = 55), and daily GH/somapacitan (n = 51) (Fig. 3). Sixteen patients who received daily GH withdrew before the extension period; safety results are reported for this group as ‘daily GH/no treatment’.

Baseline characteristics are shown in Table 2 and were similar among the groups. More patients had adult-onset AGHD (69.7%) than childhood-onset AGHD (30.3%). In all groups, most patients had multiple pituitary hormone deficiency.

**Table 2.** Patient Characteristics at Baseline

	Somapacitan	Daily GH	Placebo	Total
Characteristic	(N = 120)	(N = 119)	(N = 61)	(N = 300)
Mean age, years (SD)	44.6 (14.3)	45.7 (15.3)	45.0 (15.7)	45.1 (15.0)
Female, n (%)	62 (51.7)	61 (51.3)	32 (52.5)	155 (51.7)
Race, n (%)				
White	82 (68.3)	76 (63.9)	42 (68.9)	200 (66.7)
Asian	34 (28.3)	36 (30.3)	16 (26.2)	86 (28.7)
Black or African American	2 (1.7)	3 (2.5)	2 (3.3)	7 (2.3)
Other/NA	2 (1.7)	4 (3.4)	1 (1.6)	7 (2.3)
Mean body weight, kg (SD)	76.2 (21.0)	76.0 (22.7)	69.8 (19.7)	74.8 (21.5)
Mean BMI, kg/m^2^ (SD)	27.9 (6.3)	27.7 (6.2)	26.1 (6.4)	27.4 (6.3)
Mean waist circumference, cm (SD)	93.9 (16.6)	94.3 (15.1)	88.2 (14.5)	92.9 (15.7)
Mean IGF-I SDS (SD)	–2.58 (1.21)	–2.53 (1.18)	–2.68 (1.29)	NR
GHD onset, N (%)				
Childhood–idiopathic	21 (17.5)	21 (17.6)	13 (21.3)	55 (18.3)
Childhood–organic	17 (14.2)	12 (10.1)	7 (11.5)	36 (12.0)
Adulthood	82 (68.3)	86 (72.3)	41 (67.2)	209 (69.7)
Type of GHD, N				
GHD only	2	3	4	9
Multiple pituitary hormone deficiency^a^	118	116	57	291
With diabetes mellitus, n (%)	7 (5.8)	6 (5.0)	3 (4.9)	16 (5.3)

Abbreviations: BMI, body mass index; GHD, growth hormone deficiency; IGF-I, insulin-like growth factor-I; NA, not applicable; NR, not reported; SDS, standard deviation score

^a^Multiple pituitary hormone deficiency was defined as GHD plus at least one other deficiency (terms reported: panhypopituitarism, empty sella syndrome, Sheehan syndrome, single axis deficiencies), or if concomitant medication included treatment for deficiency of one or more pituitary axes.

**Figure 4.**
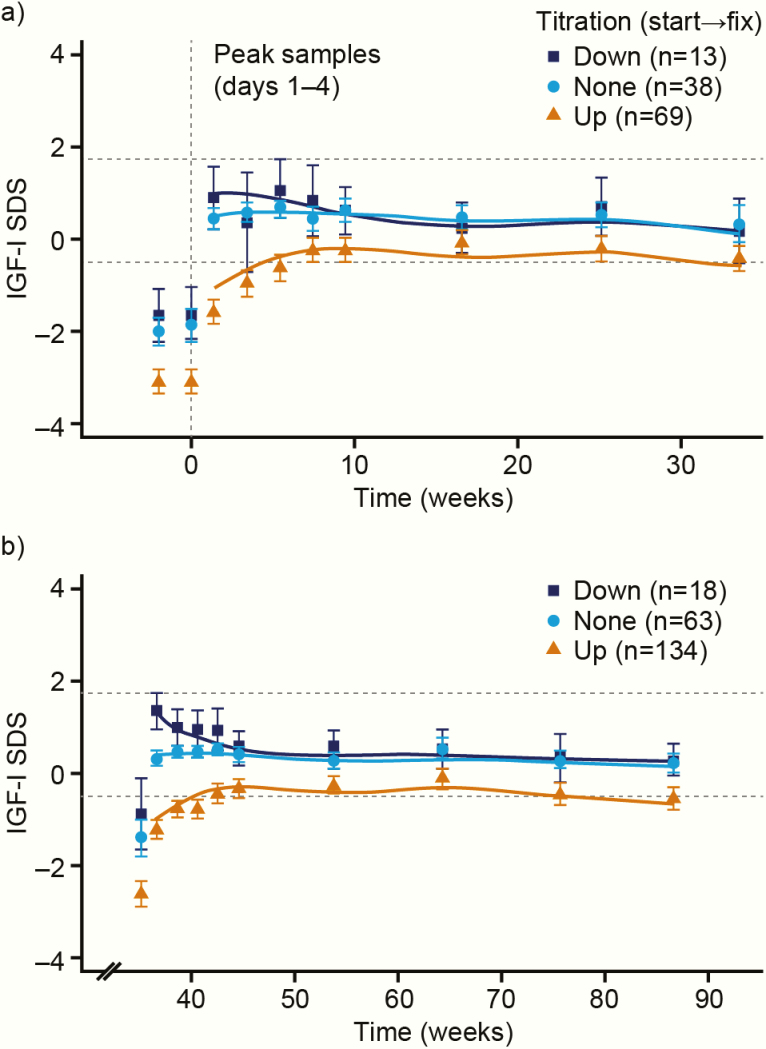
Time course of IGF-I SDS according to titration adjustments for somapacitan. (a) Main period; (b) Extension period. Peak IGF-I samples (days 1–4) by titration (down, up, or none) from start dose to fixed dose of somapacitan for subjects entering the fixed-dose period. For visual purposes, trough IGF-I samples are not included. Data are observed mean (95% CI) (points and error bars) and the mean of individual predictions (solid lines). Dotted lines show target IGF-I SDS range. Abbreviations: IGF-I, insulin-like growth factor-I; SDS, standard deviation score.

Exposure to and mean adherence with treatment are shown in Table 3. Mean IGF-I SDS values for the somapacitan group are shown in Fig. 4 according to patients’ titration from starting dose to fixed dose.

**Table 3.** Exposure to Treatment and Adherence to Treatment

*a) Main period*				
	Somapacitan	Daily GH	Placebo	
	(N = 120)	(N = 119)	(N = 61)	
Exposure to treatment, days				
Mean	232 (36.2)	226 (48.0)	228 (36.1)	
Median	238	238	238	
Treatment dose, mg, mean (SD)	2.56 (1.48) mg/week Equivalent to 0.37 mg/day	0.33 (0.19) mg/day Equivalent to 2.31 mg/week	2.20 (0.74) mg/week Equivalent to 0.31 mg/day	
Mean adherence, %	95.5	90.6	93.9	
***b) Extension period***				
	**Somapacitan/ Somapacitan**	**Daily GH/ Daily GH**	**Placebo/ Somapacitan**	**Daily GH/ Somapacitan**
	**(N** = **114)**	**(N** = **52)**	**(N** = **55)**	**(N** = **51)**
Exposure to treatment, days				
Mean	355 (52.4)	349 (48.8)	357 (40.4)	343 (76.6)
Median	364	364	364	364
Treatment dose in extension, mg, mean (SD)	2.35 (1.30) mg/week Equivalent to 0.34 mg/day	0.28 (0.16) mg/day Equivalent to 1.96 mg/week	2.61 (1.40) mg/week Equivalent to 0.37 mg/day	2.66 (1.37) mg/week Equivalent to 0.38 mg/day
Mean adherence during extension, %	94.7	91.9	89.8	94.7

### Body composition

#### Outcomes at 34 weeks.

**Figure 5.**
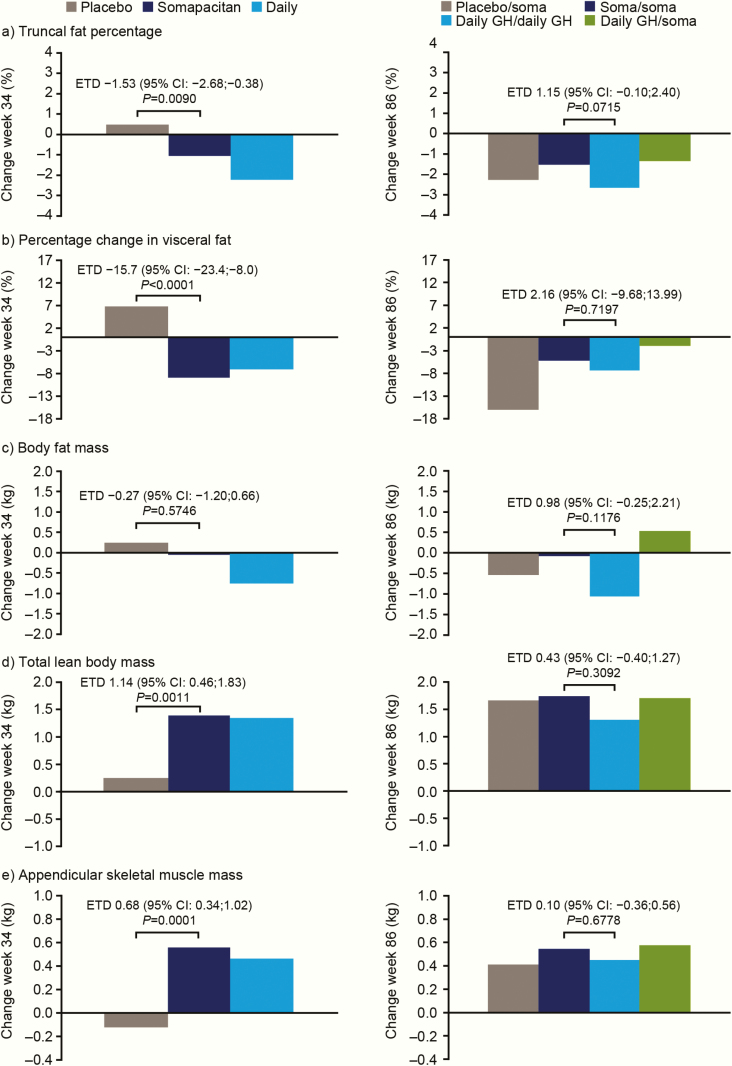
Adjusted changes from baseline in DXA-derived body composition measures during the main period (left hand graphs) and extension period (right hand graphs) (full analysis set). ETD values are shown for somapacitan minus placebo (main period) and for somapacitan/somapacitan minus daily GH/daily GH (extension period). For effects on fat mass, a reduction is desired. A negative ETD means the reduction appeared more pronounced with somapacitan than with the comparator. For effects on lean mass, an increase is desired. A positive ETD means the increase appeared more pronounced with somapacitan than with the comparator. Adjusted values are change from baseline estimates based on an analysis of covariance model (main period) or mixed model for repeated measurements (extension period) adjusted for baseline characteristics. The y-axes show the adjusted change from baseline at week 34 or week 86. Abbreviations: CI, confidence interval; DXA, dual-energy x-ray absorptiometry; ETD, estimated treatment difference; GH, growth hormone; soma, sompacitan.

A reduction in truncal fat percentage from baseline to week 34 was observed for somapacitan (−1.06%) compared with an increase for placebo (0.47%) (Fig. 5). At week 34, the estimated treatment difference (ETD) for truncal fat percentage was statistically significant (ETD [95% CI], −1.53% [−2.68; −0.38]; *P* value: 0.0090), confirming superiority of somapacitan over placebo.

Full results for the DXA endpoints are shown in Tables 4A and 4B. Visceral fat decreased from baseline to week 34 in the somapacitan group, whereas total lean body mass and appendicular skeletal muscle mass increased compared with placebo (Table 4A, Fig. 5).

**Table 4.** Baseline Values, Observed Change from Baseline to Week 34 (a) and Week 86 (b) in DXA-Derived Body Composition Measures, and Adjusted Between-Group Differences (Full Analysis Set)

*a) Main period: patients treated with once-weekly somapacitan, once-weekly placebo or daily GH*					
	Somapacitan observed mean (SD)	Daily GH observed mean (SD)	Placebo Observed mean (SD)	Adjusted between-group difference (95% CI)^a^ Somapacitan—placebo	Adjusted between-group difference (95% CI)^a^ Somapacitan—daily GH
**Effects on fat mass**					
Truncal fat percentage, %					
Baseline	39.11 (8.81)	38.10 (9.65)	36.90 (8.98)		
Change to week 34	–1.16 (2.91)	–2.47 (4.54)	+0.63 (3.17)	–1.53 (–2.68, –0.38); *P* = 0.0090	+1.17 (0.23; 2.11)^b^
Percentage change in visceral fat^c^					
Baseline	100.0	100.0	100.0		
Change to week 34	–9.41 (22.11)	–8.31 (25.46)	+6.30 (18.04)	–15.7 (–23.4; –8.0); *P* < 0.0001	–1.7 (–8.0; 4.5); *P* = 0.5817
Total fat mass, kg					
Baseline	27.56 (10.80)	27.26 (11.97)	24.82 (11.36)		
Change to week 34	–0.08 (3.05)	–0.81 (3.15)	+0.39 (2.53)	–0.27 (–1.20, 0.66); *P* = 0.5746	+0.72 (–0.04; 1.49); *P* = 0.0629
Truncal fat mass, kg					
Baseline	14.78 (6.27)	14.12 (6.32)	12.67 (6.01)		
Change to week 34	–0.18 (1.78)	–0.61 (1.89)	+0.48 (1.42)	–0.50 (–1.05; –0.06); *P* = 0.0786	+0.41 (–0.04; 0.86); *P* = 0.0749
Gynoid fat mass, kg					
Baseline	4.21 (1.67)	4.21 (1.83)	4.08 (2.17)		
Change to week 34	+0.02 (0.51)	–0.12 (0.47)	+0.01 (0.55)	+0.02 (–0.14; 0.17); *P* = 0.8511	+0.15 (0.02; 0.28); *P* = 0.0276
Android fat mass, kg					
Baseline	24.80 (1.22)	23.95 (1.23)	20.89 (1.11)		
Change to week 34	–0.08 (0.36)	–0.15 (0.32)	+0.06 (0.27)	–0.12 (–0.22; –0.01); *P* = 0.0326	+0.07 (–0.01; 0.16); *P* = 0.0923
**Effects on lean mass**					
Total lean body mass, kg					
Baseline	45.48 (13.11)	45.66 (14.32)	42.53 (11.01)		
Change to week 34	+1.38 (2.12)	+1.44 (2.29)	+0.31 (2.09)	1.14 (0.46, 1.83); *P* = 0.0011	0.05 (–0.51; 0.61); *P* = 0.8653
Truncal lean body mass, kg					
Baseline	22.30 (6.64)	22.46 (7.34)	20.70 (5.57)		
Change to week 34	+0.79 (1.37)	+0.89 (1.35)	+0.41 (1.26)	0.45 (0.03, 0.88); *P* = 0.0380	–0.04 (–0.39; 0.31); *P* = 0.8295
Appendicular skeletal muscle mass, kg					
Baseline	20.30 (6.58)	20.35 (7.02)	18.96 (5.49)		
Change to week 34	+0.56 (1.01)	+0.51 (1.25)	–0.12 (1.01)	0.68 (0.34, 1.02); *P* = 0.0001	0.10 (–0.18; 0.37); *P* = 0.4996
***b) Extension period: patients treated with somapacitan/somapacitan versus daily GH/daily GH. Note that baseline refers to the beginning of the trial and not to week 34***					
	**Somapacitan/ somapacitan** **Observed mean (SD)**	**Daily GH/daily GH** **Observed mean (SD)**	**Placebo/ somapacitan** **Observed mean (SD)**	**Daily GH/ somapacitan** **Observed mean (SD)**	**Adjusted between-group difference at week 86 (95% CI)** ^**d**^ ** Somapacitan/ somapacitan –** **Daily GH/daily GH**
**Effects on fat mass**					
Truncal fat percentage, %					
Baseline	39.12 (8.81)	38.16 (9.46)	37.00 (8.89)	38.26 (9.59)	
Change to week 86	–1.68 (3.64)	–2.77 (4.69)	–2.16 (3.94)	–0.96 (4.51)	1.15 (–0.10;2.40); *P* = 0.0715
Percentage change in visceral fat^c^					
Baseline	100.00	100.00	100.00	100.00	
Change to week 86	–5.41 (28.29)	–5.84 (28.66)	–16.09 (35.20)	–0.88 (38.42)	2.16 (–9.68;13.99); *P* = 0.7197
Total fat mass, kg					
Baseline	27.56 (10.80)	27.97 (11.89)	24.86 (11.34)	26.82 (12.01)	
Change to week 86	–0.20 (3.72)	–1.01 (4.14)	–0.54 (3.25)	+0.88 (4.79)	0.98 (–0.25;2.21); *P* = 0.1176
Truncal fat mass, kg					
Baseline	14.78 (6.27)	14.50 (6.23)	12.72 (6.00)	14.00 (6.68)	
Change to week 86	–0.25 (2.22)	–0.76 (2.27)	–0.31 (1.77)	+0.36 (2.55)	0. 61 (–0.09; 1.30); *P* = 0.0876
Gynoid fat mass, kg					
Baseline	4.21 (1.66)	4.30 (1.86)	4.05 (2.16)	4.17 (1.81)	
Change to week 86	+0.003 (0.56)	–0.12 (0.63)	–0.09 (0.76)	+0.14 (0.79)	0.14 (–0.07; 0.35); *P* = 0.1839
Android fat mass, kg					
Baseline	2.48 (1.22)	2.43 (1.16)	2.09 (1.10)	2.40 (1.30)	
Change to week 86	–0.05 (0.47)	–0.12 (0.38)	–0.11 (0.37)	+0.01 (0.49)	0.10 (–0.04; 0.24); *P* = 0.1679
**Effects on lean mass**					
Total lean body mass, kg					
Baseline	45.48 (13.11)	46.14 (12.75)	42.49 (10.97)	44.68 (15.21)	
Change to week 86	1.73 (2.53)	1.50 (2.48)	1.72 (2.97)	1.68 (3.41)	0.43 (–0.40;1.27); *P* = 0.3092
Truncal lean body mass, kg					
Baseline	22.29 (6.64)	22.77 (6.37)	20.68 (5.55)	21.97 (7.88)	
Change to week 86	1.15 (1.49)	1.00 (1.28)	1.20 (1.50)	1.02 (1.55)	0.27 (–0.18;0.72); *P* = 0.2421
Appendicular skeletal muscle mass, kg					
Baseline	20.31 (6.58)	20.45 (6.35)	18.96 (5.49)	19.99 (7.44)	
Change to week 86	0.54 (1.23)	0.48 (1.53)	0.45 (1.69)	0.63 (1.93)	0.10 (–0.36;0.56); *P* = 0.6778

Abbreviations: CI, confidence interval; DXA, dual-energy x-ray absorptiometry

^a^Adjusted values based on analysis of covariance model. ^b^This was not designed as a confirmatory test and no hierarchical test strategy was constructed; therefore, no p value was calculated. ^c^*Post hoc*-defined endpoint. ^d^Adjusted data based on mixed model for repeated measurements. Changes are expressed as a percentage, therefore initial values are 100%.

Improvements indicating desired changes in body composition were seen with both somapacitan and daily GH for the following parameters: truncal fat percentage, percentage change in visceral fat, total fat mass, truncal fat mass, total lean body mass, and appendicular skeletal muscle mass at 34 weeks (Table 4A). Based on the changes from baseline, a less pronounced reduction in truncal fat percentage, a similar reduction in visceral fat, and similar increases in total lean body mass and appendicular skeletal muscle mass were observed with somapacitan compared with daily GH.

#### Outcomes at 86 weeks.

Beneficial changes in overall body composition were observed after 86 weeks of treatment with both once-weekly somapacitan and daily GH (Table 4B, Fig. 5).

After 86 weeks of continuous treatment with somapacitan, the adjusted change from baseline in truncal fat percentage was −1.52% (Fig. 5).

There were no notable differences between somapacitan and daily GH for percentage change in visceral fat, lean body mass, or appendicular skeletal muscle mass after 86 weeks of treatment. In both groups, a reduction in visceral fat and increases in total lean body mass and appendicular skeletal muscle mass were maintained (Table 4B, Fig. 5). The effects after 86 weeks of treatment on the remaining fat body composition parameters remained less pronounced with somapacitan compared with daily GH (Table 4B).

In the group who switched from placebo to treatment with somapacitan after week 34, improvements were observed across all body composition parameters after 52 weeks of treatment with somapacitan (Table 4B, Fig. 5). These improvements were of a similar magnitude to the effects induced by daily GH.

#### Bone parameters.

Mean levels in corrected total bone mineral content and corrected total bone mineral density were unchanged from baseline to week 86, both with somapacitan and daily GH, and no differences between the somapacitan/somapacitan and daily GH/daily GH groups were observed.

#### Metabolic and inflammatory markers.

At week 34, adjusted changes in body weight from baseline to week 34 were small and did not differ between somapacitan (+1.40 kg) and placebo (+0.39 kg) but differed between somapacitan and daily GH (+0.27 kg; ETD 1.13 [0.13; 2.12]). There were no changes from baseline to week 34 in any of the treatment groups, or differences between treatment groups, in waist circumference, lipid profile, hsCRP, or interleukin-6.

At week 86, changes in body weight from baseline were small and did not differ between somapacitan/somapacitan and daily GH/daily GH. No change was observed in the estimated mean waist circumference from baseline to week 86 in the somapacitan/somapacitan arm (0.65 cm), whereas a decrease was observed in the daily GH/daily GH arm (−1.88 cm) (ETD 2.53 [0.6; 4.5]). There were no changes from baseline to week 86 in lipid profile, hsCRP, or interleukin-6.

### IGF-I SDS and IGFBP-3 SDS concentrations

#### IGF-I SDS and IGFBP-3 SDS at 34 weeks.

**Figure 6.**
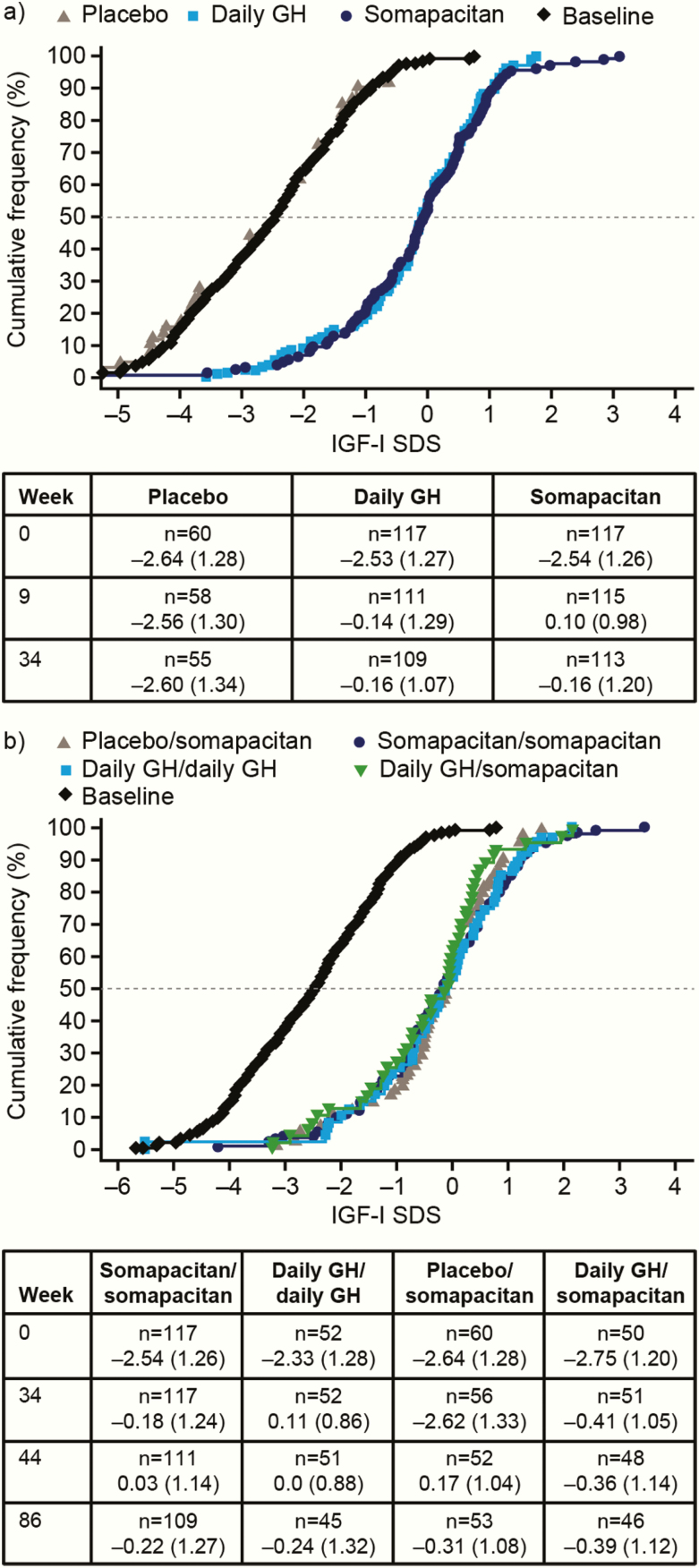
Empirical distribution (cumulative frequency) of IGF-I SDS values. Mean values at specific timepoints are shown in the tables below the figures. (a) Main period: distribution at week 34. Baseline values are also shown. IGF-I SDS increased in the somapacitan and daily GH groups but not in the placebo group. (b) Extension period: distribution at week 86. IGF-I SDS increased in all treatment groups. The black curve shows baseline values. Weeks 9 (main period) and 44 (extension period) mark the first visit after the end of the titration period. Full analysis set. n values show the number of patients contributing data (IGF-I values were not available for all patients at all visits). Abbreviations: GH, growth hormone; IGF-I, insulin-like growth factor-I; SDS, standard deviation score.

At baseline, mean IGF-I SDS was below −2.5 in all groups (Table 2). Mean serum IGF-I SDS increased markedly after 34 weeks of treatment with somapacitan and daily GH, to similar values (ETD, 0.02 [−0.23; 0.28]), but did not increase with placebo (ETD somapacitan—placebo: 2.40; 95% CI, 2.09, 2.72) (Fig. 6A). A similar pattern was observed for serum IGFBP-3 SDS (data not shown).

Seven patients (somapacitan) and 4 patients (daily GH) had transient IGF-I SDS values above +2 at one or more time points during the fixed-dose treatment period (weeks 9–34).

#### IGF-I and IGFBP-3 at 86 weeks.

Mean observed serum IGF-I SDS did not change markedly between weeks 34 and 86, except for patients who switched from placebo to somapacitan (Fig. 6B). In these patients, IGF-I SDS increased from −2.62 (1.33) to −0.31 (1.08). A similar response was observed for IGFBP-3. No treatment differences in change from baseline to 86 weeks in IGF-I SDS and IGFBP-3 SDS were observed between somapacitan and daily GH (ETD in IGF-I SDS for somapacitan/somapacitan minus daily GH/daily GH group: 0.07 [−0.29; 0.44]).

During the fixed-dose treatment period of the extension (weeks 44–86), patients had transient IGF-I SDS value above +2, as follows: somapacitan/somapacitan n = 9, daily GH/daily GH n = 2, placebo/somapacitan n = 6, and daily GH/somapacitan n = 3.

### Safety

#### Adverse events.

For the trial period overall (weeks 1–87), AEs occurred at the following rates per 100 patient-years at risk: somapacitan/somapacitan 359.3, daily GH/daily GH 442.5, placebo/somapacitan 416.5, daily GH/somapacitan 448.9, and daily GH/no treatment 542.0. Serious AEs (SAEs) occurred in patients as follows: somapacitan/somapacitan, 13 patients (10.8%); daily GH/daily GH, 5 (9.6%); placebo/somapacitan, 8 (13.1%); daily GH/somapacitan, 7 (13.7%); and daily GH/no treatment, 4 (25.0%). Of 68 SAEs reported in the trial overall, 2 were judged as probably/possibly related to trial products. An SAE of hemoconcentration in the daily GH/no treatment group referred to above was judged as probably related to daily GH by the investigator; this patient was also receiving testosterone (a known cause of hemoconcentration in male hypopituitary patients) for gonadotrophin deficiency. This SAE was also reported as life-threatening, but the patient recovered after being treated. A second SAE, a bladder transitional cell carcinoma diagnosed in week 80 during somapacitan treatment in the daily GH/somapacitan group, was assessed as possibly related to both daily GH and somapacitan. The patient completed the trial, in agreement with the protocol, and the final outcome was recorded as not recovered and chronic.

During the main treatment period, 1 death was reported: a 76-year-old patient in the placebo group died due to acute adrenocortical insufficiency, regarded as unrelated to the study drug. During the extension period, 4 deaths were reported: 1 each in the daily GH/daily GH, placebo/somapacitan, daily GH/somapacitan, and daily GH/no treatment groups; all were deemed unlikely to be related to study treatment.

**Figure 7.**
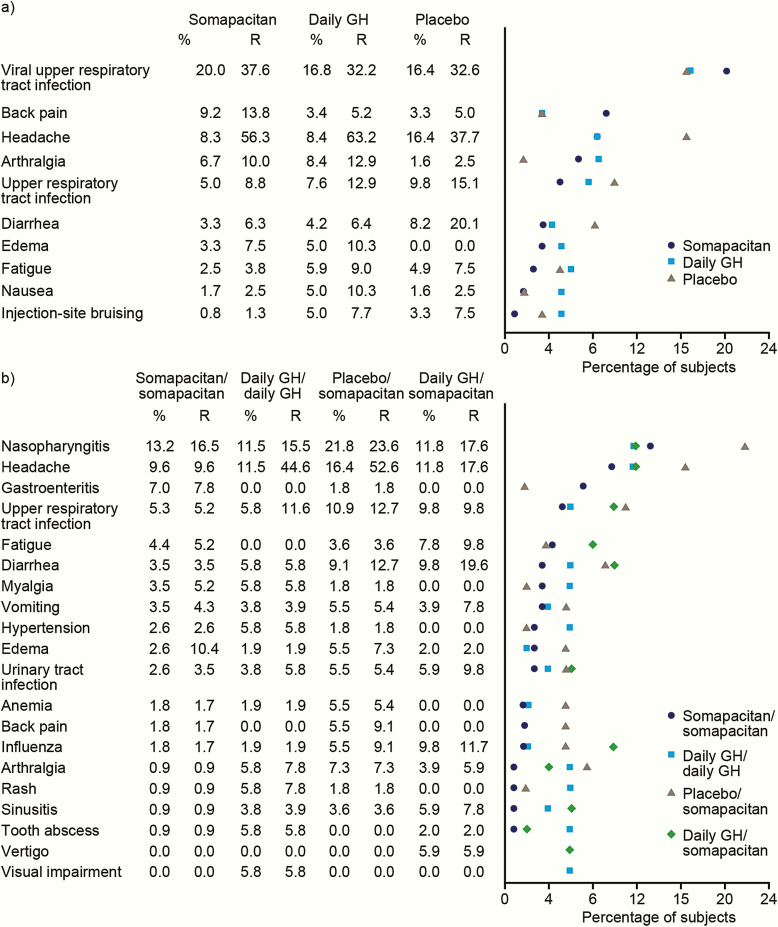
Most frequent adverse events, occurring in ≥5% of patients in any treatment arm. (a) Main period. (b) Extension period. Abbreviations: %, Percentage of exposed subjects having the event; R, event rate per 100 patient-years at risk.

During the main period, AEs occurred in 72.5%, 79.8%, and 75.4% of patients in the somapacitan, daily GH, and placebo groups, respectively. The majority of AEs were of mild/moderate severity and reported as unlikely to be related to the study drugs. The most frequent AEs (≥ 5%) included upper respiratory tract infections, headache, back pain, and arthralgia across treatment groups (Fig. 7A). SAEs occurred in 7 (5.8%), 11 (9.2%), and 5 (8.2%) patients in the somapacitan, daily GH, and placebo groups, respectively.

An overview of AEs during the main and extension periods for patients treated throughout with somapacitan or daily GH is presented in Table 5. During the extension period, rates of AEs per 100 patient years at risk were 270.5 (somapacitan/somapacitan) and 333.6 (daily GH/daily GH) (Table 5). The most frequently reported AEs with start dates during the extension period included nasopharyngitis, headache, gastroenteritis, and upper respiratory tract infections (Fig. 7b).

**Table 5.** Overview of Adverse Events in the Main and Extension Periods for Patients Treated Throughout with Somapacitan or Daily GH.

	Reported in main period (34 weeks)								Reported in extension period (52 weeks)							
	Somapacitan				Daily GH				Somapacitan/somapacitan				Daily GH/daily GH			
	N	(%)	E	R	N	(%)	E	R	N	(%)	E	R	N	(%)	E	R
**Number of subjects exposed**	**120**				**119**				**114**				**52**			
**All events**	**87**	**(72.5)**	**385**	**482.0**	**95**	**(79.8)**	**426**	**549.4**	**77**	**(67.5)**	**311**	**270.5**	**41**	**(78.8)**	**172**	**333.6**
Serious events	7	(5.8)	12	15.0	11	(9.2)	13	16.8	7	(6.1)	12	10.4	3	(5.8)	5	9.7
Mild events	75	(62.5)	273	341.8	81	(68.1)	280	361.1	71	(62.3)	223	194.0	35	(67.3)	106	205.6
Moderate events	34	(28.3)	101	126.4	42	(35.3)	136	175.4	30	(26.3)	78	67.8	21	(40.4)	62	120.3
Severe events	7	(5.8)	11	13.8	9	(7.6)	10	12.9	6	(5.3)	10	8.7	2	(3.8)	4	7.8
**Relationship**																
Probably related	14	(11.7)	23	28.8	14	(11.8)	24	31.0	6	(5.3)	10	8.7	2	(3.8)	2	3.9
Possibly related	23	(19.2)	70	87.6	27	(22.7)	51	65.8	16	(14.0)	31	27.0	6	(11.5)	10	19.4
Unlikely related	79	(65.8)	292	365.6	89	(74.8)	351	452.7	74	(64.9)	270	234.9	39	(75.0)	160	310.4

#### Injection-site reactions.

Injection-site reactions were reported as follows: somapacitan 8 (6.7%) patients, 12 events; daily GH 7 (5.9%) patients, 10 events; and placebo 6 (9.8%) patients, 11 events during the main period. During the extension period, injection-site reactions were reported as follows: somapacitan/somapacitan, 2 (1.8%) patients, 3 events; daily GH/daily GH, 2 (3.8%) patients, 3 events; placebo/somapacitan, 1 (1.8%) patient, 1 event; daily GH/somapacitan, 3 (5.9%) patients, 6 events. All injection-site reactions during both periods were evaluated as mild or moderate.

#### Glucose metabolism.

**Figure 8.**
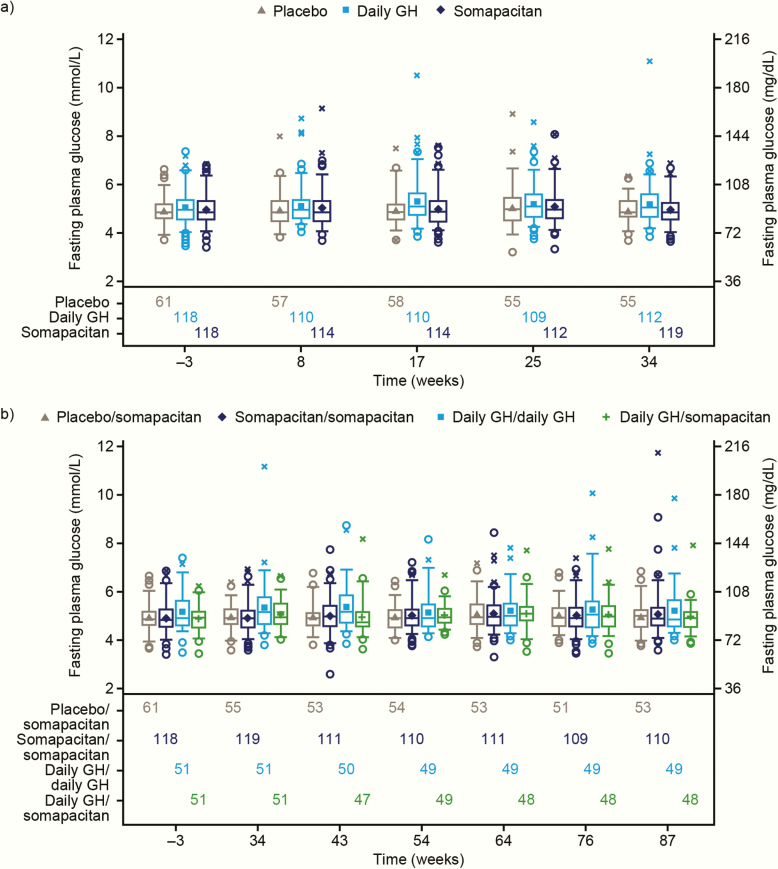
Fasting plasma glucose by visit (safety analysis set). (a) Main period. (b) Main and extension periods. Observed data. Mean (filled symbols), median (center line), 25th and 75th percentiles (box), 5th and 95th percentiles (whiskers), individual outliers: nondiabetic at baseline (open circles), diabetic at baseline (crosses). Numbers of patients contributing to the data points appear in the bottom panel. Abbreviations: FPG, fasting plasma glucose.

Only minor changes in mean fasting plasma glucose (FPG) (Fig. 8) or HbA_1c_ were observed from baseline to end of trial in any treatment arms in either period. At the end of the trial, similar mean FPG values were observed across 4 treatment arms, shown as mmol/L (mg/dL): somapacitan/somapacitan, 5.06 (91.1); daily GH/daily GH, 5.20 (93.6); placebo/somapacitan, 4.98 (89.6); daily GH/somapacitan, 4.95 (89.1). Changes from baseline in mmol/L (mg/dL) were +0.11 (2.0), +0.05 (0.9), +0.06 (1.1), and +0.07 (1.3), respectively. Mean HbA_1c_ (%) values were 5.52, 5.50, 5.55, and 5.48, and changes from baseline were +0.10, +0.11, +0.09, and +0.07, respectively. In total, 9 patients (somapacitan/somapacitan, 4; daily GH/daily GH, 2; placebo/somapacitan, 2; daily GH/somapacitan, 1) had FPG increased from normal at baseline to high during the course of the trial. Two patients (both treated with daily GH) were diagnosed with diabetes mellitus during the trial; there were no new cases of diabetes among patients treated with somapacitan.

#### Antibody assessments.

No anti-somapacitan antibodies were detected. Transient non-neutralizing anti-human GH antibodies were detected in 1 patient from the daily GH group at the 4-week visit only.

## Discussion

In patients with AGHD, somapacitan administered once weekly demonstrated superiority over placebo in reducing truncal fat percentage. Somapacitan also reduced visceral fat and increased total lean body mass and appendicular skeletal muscle mass. These beneficial changes were maintained for up to 86 weeks of treatment. Somapacitan was well-tolerated over 86 weeks and there were no unexpected safety findings.

The reduction in visceral fat observed with somapacitan at 34 and 86 weeks is notable because visceral fat is an important indicator of metabolic risk, in particular the risk of cardiovascular disease and type 2 diabetes (23). Other studies have shown that the reduction in visceral fat in response to GH treatment is larger than the reduction in subcutaneous fat and total body fat (24). Increases in lean body mass have been shown in previous studies to be associated with increased muscle strength in AGHD (25).

In addition to the double-blind comparison to placebo, the trial also included a comparison with open-label daily GH, to allow clinical judgment of the effects in comparison with standard-of-care GH replacement therapy in AGHD patients. At 34 weeks, a less pronounced reduction in truncal fat percentage, a similar percentage reduction in visceral fat, a similar increase in total lean body mass, and a more pronounced increase in appendicular skeletal muscle mass were observed with somapacitan compared with daily GH. Overall, therefore, the beneficial changes in body composition induced by somapacitan were in line with the known effects of GH replacement in adult patients with GHD (a decrease in body fat, particularly visceral fat, and increases in lean/muscle mass) (26).

An important subgroup to consider during the extension period is those patients initially randomized to placebo who switched to somapacitan after 34 weeks. They represent a second group of patients naïve to GH or not taking it for at least 6 months, just as with the initial group randomized to placebo for the main study. This second cohort experienced improvements across all body composition parameters that were of a similar magnitude to those induced by daily GH. These similar effects of somapacitan seen in 2 separate groups of patients support the benefit on body composition and an overall similar treatment effect of somapacitan and daily GH.

The reduction in visceral fat and the increases in total lean body mass and appendicular skeletal muscle mass were maintained in all the active treatment groups at 86 weeks. In the patients continuing on once-weekly somapacitan in the 52-week extension, the effects after 86 weeks of treatment on the remaining fat mass parameters remained less pronounced with somapacitan compared with daily GH. However, as mentioned above, in patients switched from placebo to somapacitan, the effects on body composition were similar to those who received daily GH throughout. Thus, taken together, an overall similar treatment effect of somapacitan and daily GH was observed.

The less pronounced effect of somapacitan compared with daily GH observed for the reduction in some of the adipose tissue parameters after 34 weeks of treatment could not be explained by a different response in IGF-I SDS, as mean IGF-I SDS levels and distribution of IGF-I SDS were similar between treatment groups at weeks 8 (end of titration) and 34 (end of main period). The number of female patients on oral estrogen differed in the treatment groups, as follows (shown as number on estrogen/number in the treatment arm) in the main period: somapacitan, n = 38/120; daily GH group, n = 23/119; placebo, n = 10/61. Continuing in the extension period, the distribution was: somapacitan/somapacitan, n = 36/114; daily GH/daily GH, n = 8/52; placebo/somapacitan, n = 9/55; daily GH/somapacitan, n = 12/51). A *post hoc* analysis was carried out to evaluate separately the effects of somapacitan, daily GH, and placebo on female patients on oral estrogen and on the entire trial population, excluding female patients on oral estrogen (data not shown). The results suggested that the more pronounced effects observed for some of the adipose tissue parameters in the entire trial population may have been influenced by this difference in numbers. Oral estrogen exposes the liver to high levels of estrogen, which has been shown to inhibit the action of GH on its receptor (27). Women receiving oral estrogen will therefore have a lower IGF-I response to GH, possibly resulting in diminished metabolic effects (e.g., on various fat compartments).

Blood samples for dose titration were taken 3 days after a dose of somapacitan, when IGF-I SDS values could be expected to be close to their maximum. The apparently slightly higher number of transient elevations in IGF-I SDS to above +2 in the somapacitan-treated patients reflects the larger IGF-I peak-to-trough ratio seen with somapacitan compared with daily GH, which means that IGF-I SDS values based on single measures cannot be directly compared. Pharmacokinetic/pharmacodynamic modeling has shown that *average* IGF-I levels over 1 week after dose titration are predicted to be similar between somapacitan and daily GH (17, 28).

The planned targeted steady state IGF-I SDS (between −0.5 and +1.75) was achieved for the mean IGF-I SDS in all active treatment arms in both periods. Mean IGF-I SDS values were similar for somapacitan and daily GH at the end of treatment in both periods. Mean IGF-I SDS was −0.16 (somapacitan and daily GH groups) at the end of the main period, and between −0.39 and −0.22 at the end of the extension. These values may reflect the relatively low target range, which was defined in order to keep IGF-I SDS values below +2. However, some patients in the active drug groups had IGF-I SDS values below −2 at the end of the titration period, suggesting that the titration schedule may not have allowed enough time or titration steps for these patients to reach an optimal dose.

It has been questioned whether a more continuous exposure of GH will elicit similar responses to normal physiological pulsatile exposure. However, an earlier study showed that continuous subcutaneous infusion versus daily subcutaneous injections of GH for 6 months had a similar impact on the IGF-IGFBP axis, body composition, bone metabolism, and insulin sensitivity, suggesting similar efficacy (29).

One of the concerns about continuous exposure to GH is that it could result in worsening of insulin sensitivity and glucose metabolism. FPG and HbA_1c_ values did not, however, increase over 86 weeks, and there were no new cases of diabetes in the subjects treated with somapacitan, in line with results from an earlier 26-week trial of once-weekly somapacitan versus daily GH (18). These data are also in line with results from previous studies of long-acting GH preparations (9, 10).

No new or unexpected safety concerns were reported. Reported AEs were events well known to occur during GH treatment, and their incidence and severity in patients treated with once-weekly somapacitan were similar to those in patients treated with once-weekly placebo or daily GH. Only a few, mild-to-moderate injection-site reactions were reported, with no notable differences between somapacitan and daily GH. No anti-somapacitan antibodies or neutralizing anti-GH antibodies were detected in patients treated with somapacitan. The dropout rate in the trial was low, and adherence with medication was high in all treatment groups.

Strengths of this trial include having an active comparator as well as a placebo arm, and the large number of patients involved, in 17 countries over 5 continents. This is, to our knowledge, the largest randomized controlled trial ever conducted in AGHD to compare 2 different treatments or treatment versus placebo. It is also the longest randomized trial of a long-acting GH derivative.

However, this trial had some limitations. The trial was designed and powered as a superiority trial against placebo, and not for detecting differences between somapacitan and daily GH. Patients receiving daily GH were not blinded and were therefore aware throughout the trial that they were receiving an effective drug, which could have had an impact on outcome. Not all patients reached the IGF-I SDS target; thus, some patients may not have achieved the full benefit of GH replacement on body composition.

In conclusion, somapacitan administered once weekly demonstrated superiority over placebo in patients with AGHD. The overall treatment effects of somapacitan were in accordance with well-recognized effects of GH replacement, with a reduction in abdominal fat mass and an increase in skeletal muscle mass. Somapacitan may provide an alternative to daily GH for these patients, with the need for less frequent injections expected to reduce burden of treatment and cause less interference with daily life.

## Abbreviations

AE: adverse event
AGHD: adult growth hormone deficiency
ANCOVA: analysis of covariance
DXA: dual-energy x-ray absorptiometry
ETD: estimated treatment difference
FPG: fasting plasma glucose
GH: growth hormone
GHD: growth hormone deficiency
GLP-1: glucagon-like peptide-1
HbA_1c_: glycated hemoglobin A_1c_
hsCRP: high-sensitivity C-reactive protein
IGF-I: insulin-like growth factor 1
IGFBP-3: insulin-like growth factor binding protein-3
ITT: insulin tolerance test
MMRM: mixed model for repeated measurements
SAE: serious adverse event
SDS: standard deviation score

## Additional Information

***Disclosure Summary:*** G.J. has received speaker’s honoraria from Eli Lilly, Merck Serono, Novartis, Novo Nordisk, Pfizer, Otsuka and Shire and has been a consultant for AstraZeneca, Merck, Serono, Novo Nordisk, Pfizer and Shire. M.B.G. reports research grants from Camurus, Corcept, Crinetics, Ipsen, Novo Nordisk, Opko, Strongbridge and Teva. I.H.H., M.H.R. and C.S. are employees of Novo Nordisk and all own shares in the company. W.K. has been a consultant for Eli Lilly and Novo Nordisk. S.T. has nothing to declare. K.T. has received consulting honoraria from Teijin Pharma and Novo Nordisk. B.M.K.B. has been the PI of research grants to Massachusetts General Hospital from Novo Nordisk and Opko and has received occasional consulting honoraria from Merck Serono, Novo Nordisk and Pfizer.

***Data Availability:*** The study protocol and redacted clinical study reports are available according to Novo Nordisk data-sharing commitments. The data will be available as redacted study reports permanently after research completion and approval of product use in both the EU and USA with no end date. Data will be shared with bona fide researchers submitting a research proposal requesting access to data, for analyses in line with the aims of the study protocol and approved by the Independent Review Board according to the IRB charter (see novonordisk-trials.com). Request proposal forms and the access criteria can be found at novonordisk-trials.com. The data will be made available on a specialized SAS data platform.

## List of investigators and sub-investigators

**Australia:** B Stuckey, N Palmer, G Smith, N Opie. M McLean, A Duke, J Lin. R Simpson, C Gilfillan, S Goh, D Davidoff, F Gunawan, JY Mah, M Ebrahim. P Colman, A Nankervis, S Fourlanos. L Tacon, V Tsang, C Girgis. A McCormack, N Sheriff, M Isaacs, N Manzoor. K Ho, V Chikani, A Russell. S Linjawi, D Martin.

**Germany:** H Biering, S Valentine. M Droste, G Wenzel. W Karges, C Streetz-van der Werf, S Kacheva. A Mann, V Böttcher.

**Israel:** N Stern, Y Sofer, M Serebro, Y Greenman, E Osher.

**India:** A Majumder, D Sanyal, S Roy C. N Bhagwat, Pr Varthakavi, AS Joshi. BK Sethi, M Ali, JV Kelwade. RN Mehrotra, RDPV Pavithran, H Kumar, MT Shenoy, N Abraham.

**Japan:** H Miyoshi, A Nakamura, H Nomoto, A Miyoshi, K Yamamoto, C Yamamoto, A Miya, N Kitao, R Hashimoto, K Takahashi, H Sugawara, M Dannoura, C Shimizu, KY Cho, S Yanagiya, K Tsuchida, Takase, Y Kameda, K Kito, K Omori, M Oita, S Edagawa. S Nagai, N Yoshioka, E Hirata, Y Miyano, I Shigesawa. T Tanaka, T Yoshida, H Koide, S Suzuki, N Hashimoto, H Nagano, I Sakuma, T Takiguchi, A Shiga, E Komai, T Kohno, A Tamura. S Yamada, Y Takeuchi, H Nishioka, N Fukuhara, M Okada, A Takeshita, H Suzuki, K Tastushima. S Tahara, E Ishisaka, K Hironaka, Y Hattori, K Oyama, H Ten, T Higuchi, T Kitamura, T Nozaki, Y Igarashi, K Yui, E Baba, Y Fujiki, A Kubota, D Morimoto, K Oda, S Sato. K Takano, A Hayashi, S Chida, E Kishihara, R Ichikawa, T Masaki, R Arai. T Tagami, A Shimatsu, T Usui, M Kakita, R Nakatani, M Tsuiki, H Umakoshi, T Ogasawara, K Hiroshima, M Yokomoto. M Nomura, K Ashida, S Sakamoto, H Kawate, E Terada. W Kameda, Y Hada, K Tada, K Ishii. F Otsuka, N Yamauchi, K Toma, T Hosoya. K Arita, S Fujio, H Yonezawa, H Uchida, M Habu, Y Nagano. A Matsuno, Y Ishii, T Hirohata. M Omura, J Saito, T Nishikawa, C Sugisawa, Y Tsurutani, A Suzuki. N Miki, M Ono, T Hori.

**Lithuania:** R Laukiene, A Abraitiene, R Juskiene. D Velickiene, R Knispelis, L Barsiene.

**Latvia:** V Pirags, K Geldnere, R Ritenberga, S Steina, I Balcere.

**Malaysia:** FTH Sieng, TC Ting. Z Hussien, AB Nasruddin. KC Hui, LS Lin.

**Poland:** P Witek, W Kapusta, A Styk, E Deptuła-Krawczyk. M Korpal-Szczyrska, D Birkholz-Walerzak, A Alska, M Myśliwiec. J Kuleta, B Kmak-Balawender. E Franek, K Jedynasty, EK Wojtachnio, D Kołakowska. S Daniluk, A Bołtryk, M Ruszkowska, NA Nowak. M Bolanowski, A Jawiarczyk-Przybyłowska, E Kubicka, K Zawadzka, J Jakubowska.

**Romania:** I Zosin, M-M Vlad, M Balas, I Golu, D-G Amzar. C Vulpoi, C Cristea, A-D Belceanu. CE Gerogescu, IR Ilie, CA Silaghi, S Naumescu, I Duncea. IM Pascanu, RM Pop. CV Badiu, ML Gheorghiu, C Stancu, A Gogoi. M Stanciu, IG Totoian.

**Russia:** L Rozhinskaya, A Shishkina, N Molitvoslova, Z Belaya, E Pigarova, E Przhiyalkovskaya, S Arapova. Y Samoilova, I Soboleva, O Oleynik, M Koshmeleva, E Kharlamova, M Rotkank. V Peterkova, T Shiryaeva, E Nagaeva, A Emeliyanov. G Vagapova, L Orlova, F Nasibullina, G Kashapova. M Kovarenko, O Pilipenko, E Gushchina, L Lebedeva, E Raskova. M Pavlova, K Raskina, T Tselovalnikova.

**Sweden:** G Johannsson, P Trimpou. BE Engström, Karin C Larsson.

**Turkey:** F Aral, AK Üzüm. P Kadıoğlu, D Eyice, Ö Haliloğlu, ÖP Korkmaz, HM Özkaya. Z Cantürk, ÖZ Akyay, B Çetinarslan, A Selek, İ Tarkun. T Tetiker, GT Akkuş.

**Ukraine:** O Bolshova, M Tronko, O Vyshnevskaya, O Samson, T Malinovskaya, T Marchenko, V Orlenko.

**UK:** C Higham, C Mumby, O Taha, S Adam, T Ahern. A Brooke, M Ramell. T Sathyapalan, J Zeesham, A Al-Qaissi. H Randeva, E Kyritsi, M Mytilinaiou, G Dimitriadis.

**USA:** BMK Biller, KJP Liebert. PU Freda, A Kuker, C Reyes-Vidal. M Marcelli, S Mediwala, AR Jones. MB Gordon, P Mareda, A Minnock, H Moturi, K Spiller. A Heaney, R Yu. L Kennedy, G Dobri, B Heaton, R McCoy, L Olansky, T Tomaro, D Crowe. S Nakhle, R Bowers, T Dodson, C Hyderkhan, SA Klugh, E Neylon, BM Palal, S Poynor, ASR Rosario, NJ Vernetti. HC Shea, E (Hatfield) Shelton, S Lakhian, L Olbrych. PJ Snyder, J Kharlip, EM Markmann. LD Stonesifer, C (Heins) Stock, M Miller, T Lovie-Yokum. AG Wynne, C Burns, JL Casey, F Correa, L Frazee, GG Graf, DK Soetaert. LG Abbott, C Burns, J Lybarger. J Silverstein, V Adjovu, T Bell, S Hurst, K Sterl. RJ Anderson, C Desouza, S Flor, M Gerety, V Shivaswamy, S Wagstaff. Z Litvack, K Yuen, A Brown, J Mercado, K Moloney. DG Robertson, K Dukes, S Kies. SF Rovner, BA Marranzini, VM Stewart-Ray. J Vadakekalam, DS Denham, RM Gomez, MK Korte, R Sosa, B Urrabazo. JCH Reed III, SM Estes, DD Jester, M Munoz, S Rowe, T Sapp, JM Tapia. M Welch, G Danet, N Gongora, D Harter, D Katselnik, E Leal, A Wentworth-Kotara. N Kakumanu, M Chaps, M Hebdon, J VanArsdall. JD Carmichael, N Bose, M Chiu, G Zada. J Kerr, K Kiseljak-Vassiliades, M Wierman. M Fleseriu, J Cetas, S Hopkins, STD Lim, D Ono, C Yedinak, E Varlamov. A Drincic, R Baum, W Goldner, L Kuechenmeister, J Moeller, M Severson, R Tillson. R Salvatori, TT Brown, O Kusemiju, N Mathioudakis, S Nasatka, R Ozl, M Satyal. TB Vaughan III, K Davison, T Grimes, F Ovalle, T Perkins, AH Warriner.

**South Africa:** J Wing, D Jivan. S Bhana, B Pauly. H Oosthuizen, TJ Gray, JM Koning.
